# Regional Variation in Pharmacist Perception of the Financial Impact of Medicare Part D

**DOI:** 10.3390/pharmacy6030067

**Published:** 2018-07-17

**Authors:** Shamima Khan, Joshua J. Spooner, Harlan E. Spotts

**Affiliations:** 1 CRE Services, Inc., 1560 Broadway, Suite 812, New York, NY 10036, USA; 2 College of Pharmacy and Health Sciences, Western New England University, 1215 Wilbraham Road, Springfield, MA 01119, USA; jspooner@wne.edu; 3 College of Business, Western New England University, 1215 Wilbraham Road, Springfield, MA 01119, USA; hspotts@wne.edu

**Keywords:** Medicare Part D, pharmacy, rural pharmacy, pharmacy closure

## Abstract

The objective of this study was to perform a nationwide investigation of the financial performance of community pharmacies in the United States since the inception of Medicare Part D. A nationwide, cross-sectional survey of pharmacists was conducted in 2013. The 43-item online survey collected information about demographics, financial implications of Part D on community pharmacy and patients, provision of Medication Therapy Management (MTM) services and opinions about Medicare Part D 2010 updates. The adjusted response rate was 22.3% (419/1885). A majority of respondents (75.6%) reported a stable or increased prescription volume since 2006 but only 40.4% indicated that the financial performance of their pharmacy as either excellent or good during the same period. Owners and part-owners of rural independent pharmacies were more likely to report a below average or poor financial performance (75.0%). The provision of MTM services was not related to the financial performance of the pharmacy. Nearly half (44.7%) of pharmacy owners or part-owners indicated that they were considering selling their pharmacy, with most (94.1%) reporting that their decision to sell was due to the Part D financial pressures. However, the decision to sell was not related to the change in financial performance since 2006 or the volume of prescriptions dispensed.

## 1. Introduction

The United States Medicare system (a federally administered national health insurance plan for seniors and the disabled) was vastly changed following the passage of the Medicare Prescription Drug, Improvement and Modernization Act in 2003, resulting in the establishment of new Medicare Advantage plans, expansion of allowable contributions and employer participation in health savings accounts and the establishment of a Medicare prescription drug benefit—Medicare Part D—in January 2006 [1,2]. While many individuals enrolling in Medicare Part D (“Part D”) had prescription drug coverage through a commercial or state Medicaid plan prior to Part D’s implementation, the estimated 3.4 million enrollees who lacked previous prescription coverage experienced a significant 60% reduction in their out of pocket (OOP) payments for prescription drugs and a resultant 24% increase in medication utilization in the first year of the program [3]. Longitudinal surveys have found a high degree of satisfaction among Part D from its inception to the present time [4,5].

While Part D has provided a net benefit to enrollees and is perceived positively by many physicians [2,6], the community pharmacist—especially independent pharmacy owners—have not fared as well under Part D. From the point of initial rollout, Part D plans utilized a variety of third party prescription plan controls inherent in commercial or state Medicaid plans, namely formulary restrictions and prior authorization requirements, reduced prescription dispensing payment rates and delays in receiving payment [2,7,8,9,10,11,12,13]. Pharmacists were also tasked with addressing patient enrollment issues [8]; none of these issues had existed previously for the high-margin “cash customer” (individuals without a prescription drug benefit). The changes imparted by Part D occurred at a time when the pharmacy business environment itself was undergoing a market shift. An increasingly mature market, retail pharmacy has been evolving into a duopoly controlled by major chains in an effort to maintain financial viability through operational efficiencies [14]. The commodity-like nature of filling prescriptions and governmental and commercial insurance industry cost controls were responsible for increased financial pressure on pharmacies. While traditional chain pharmacies and pharmacies within supermarkets and mass merchandising stores were able to withstand these pressures, many independent pharmacies did not survive this period; over 1400 independent pharmacies (6% of independent pharmacies nationwide) closed between 2006 and 2010 [14,15,16,17].

In an era of low rates of payment for dispensing prescriptions (with mean dispensing fees paid by health insurers currently 28% lower than they were in 1995) [18,19], pharmacists have been advised to seek alternative revenue streams and reduce their dependence on prescription gross margins as the driver of profitability [20]. One provision of the Medicare Modernization Act was the requirement that Part D prescription drug plans and Medicare Advantage prescription drug plans provide medication therapy management (MTM) programs as part of the benefit. MTM programs were created “to assure…that covered part D drugs are appropriately used to optimize therapeutic outcomes through improved medication use and to reduce the risk of adverse events, including adverse drug interactions.” [21] (p. 2086). While the Act defines basic elements of the program and who should be targeted for services, it also gave plans a large degree of flexibility for the design and implementation of the MTM program. While the Medicare Modernization Act specifically mentioned pharmacists as a provider of MTM services, the Act does not require the provider of such services be community pharmacist; few health plans are utilizing community pharmacists to provide MTM services, opting to provide these services via telephone or mail despite evidence suggesting superior prescription drug cost savings with face to face services [22,23]. Further, the rate at which community pharmacies are reimbursed for MTM services by plans does not cover the costs of delivering the service, [24] challenging the assumption that MTM services can stabilize a pharmacy’s balance sheet.

These changing pharmacy market dynamics have significantly impacted rural pharmacies. Rural pharmacies are more likely to be independently owned [25]. Due to physician scarcity, rural pharmacists often serve as first contact providers and may be the only source of healthcare in isolated rural communities [26,27,28]. The rate of rural pharmacy closures increased following the establishment of Part D; [17] these closures may leave residents without convenient access to pharmacy services and can significantly impact the population’s ability to obtain a number of essential health services [29]. Pharmacists practicing in rural independent pharmacies work longer hours and receive less compensation than those practicing in urban or suburban areas, making the ownership of a rural independent pharmacy less appealing to recent pharmacy graduates [26,29].

The impact of Part D on community pharmacy has been studied previously [9,10,11,12,13,17,30]. While these studies demonstrated financial instability within community pharmacy since the inception of Part D, these investigations were limited to a single state or region, or were retrospective in nature. This study sought to expand previously conducted research by including chain and independent pharmacies located in rural, urban and suburban settings across the country. The purpose of this study is to perform a nationwide investigation of the financial performance of community pharmacies in the United States since the inception of Part D.

## 2. Materials and Methods

A nationwide cross-sectional survey of practicing pharmacists was conducted between April and July 2013 using an internet-based survey platform (SurveyMonkey, San Mateo, CA, USA). To ensure proper representation of practicing pharmacists, a third-party vendor was selected to provide the principal investigator (PI) with a unique count of available email addresses of pharmacists practicing in independent (1 to 3 locations) and chain (4 or more locations) pharmacies. The total number of email addresses for pharmacists practicing in independent and community pharmacies were 35,911 and 51,677, respectively. To ensure proper representation from each state and Washington DC, a sample size determination, using a confidence interval of 95% and confidence level of 3, established an ideal sample size to be 17,920 and 21,221 for pharmacists practicing in independent and chain pharmacies, respectively [31]. However, budgetary constraints allowed us to send the email broadcasts (cover email and a link to the survey instrument) to a total of 7828 pharmacists (3584 practicing in independent and 4244 practicing in chain pharmacies). These email addresses were randomly selected by the third-party vendor. Following sample selection, the third-party vendor sent the introductory invitation and survey link by email broadcast to the entire sample of 7828 with four iterations. The researchers (including the PI) did not have access to the email addresses and, as such, were unable to contact the non-responding pharmacists. However, after the first introductory email broadcast, 3 consecutive email broadcasts occurred at approximately one month intervals (April 2013 (1st broadcast), May 2013, June 2013 and July 2013), which provided pharmacists ample time to respond. Survey participation was both voluntary and anonymous.

The survey instrument was developed following a thorough literature review, the results of a focus group study and two previously published multi-state, multi-region surveys of pharmacist opinions about Part D [9,10,11]. The survey included multiple choice questions, ranked ordered questions, 5-point Likert type scales and categorical scales. As the vast majority of the questions included in this survey were selected from a previously validated instrument [10,11], no pilot testing was conducted. The final instrument consisted of a total of 43 questions in multiple categories:

1. Financial Performance of Pharmacy since 2006
2. Considerations regarding the sale of the pharmacy
3. Providing Medication Therapy Management
4. Concerns about Part D 2010 Updates

The study was approved by Western New England University (WNEU) Institutional Review Board and was funded by WNEU College of Pharmacy.

Descriptive statistics (frequency counts and percentages) were used to report demographic data, outcomes data and other variables of interest. Univariate comparisons (i.e., Chi-square [χ^2^] analyses) were used to explore relationships between respondent demographics and variables of interest. Systematic comparisons in terms of pharmacists’ primary role and geographic locations of practice were explored. The outcomes of interest reported in this publication are: the financial performance of the pharmacy since the initiation of Part D; the volume of prescriptions dispensed since the initiation of Part D and for the two years prior to the completion of the survey; the dispensing of 90-days’ supply of medications under Part D; Part D prescription switching at the time of dispensing; respondent opinions about reimbursement received; the provision of MTM; the viability of the respondent’s pharmacy; and respondent opinions about the Part D 2010 updates. All demographic data in relation to practice location were analyzed; practice locations (rural/suburban/urban) were self-reported by the participants. We specifically examined relationships between providing MTM services (which was reimbursed by at least 1 Part D plan) and other variables of interest. Subsequently, we isolated all pharmacy owners- and part-owners and conducted sub-group analyses.

The primary outcome was an excellent or good financial performance of the pharmacy since the inception of Part D. This outcome was dichotomized: pharmacists who responded that the financial performance of their pharmacy has been excellent or good were compared to pharmacists who reported that the financial performance of their pharmacy was average, below average, or poor. A logistic regression model examined the relationship between the financial performance of the respondent’s pharmacy and select demographic characteristics: practice site [‘chain & other’ versus ‘independent’], primary role as a pharmacist, percentage of prescriptions received electronically, number of years in community pharmacy practice, percentage of patients enrolled in Part D and the number of prescriptions dispensed per weekday at the primary practice site.

Statistix^®^ Version 8.2 was used to conduct all statistical analyses [32].

## 3. Results

### 3.1. Survey Responses

Surveys were distributed via email to 7828 pharmacists, with 1885 determined by the third-party vendor to have reached the intended recipient. Of these, 419 responded, yielding an adjusted response rate of 22.3%. Four respondents were excluded from analyses because they were not a pharmacist (3 respondents were pharmacy technicians, 1 was a non-pharmacist pharmacy owner); an additional 9 respondents were excluded because their primary practice site did not accept Part D plans. The final sample size was 406. More than half of the respondents (56.6%) were practicing in independent pharmacies, of which 18.1% were owners and 4.0% were part-owners (data not shown). Respondents from urban locations were more likely to be practicing at an independent pharmacy setting compared to suburban and rural respondents (*P* = 0.0003). Among the respondents, 60.5% were male, 55.7% were between the ages of 51 to 70 years of age and 74.4% had practiced in community pharmacy as Registered Pharmacists (RPh) for more than 15 years (Table 1). Nearly 30% of respondents had more than 50% of their patients enrolled in Part D; respondents in rural practice settings were more likely to reach this threshold compared to suburban and urban counterparts (*P* = 0.0268).

### 3.2. Financial Performance Since 2006

**Figure 1:**
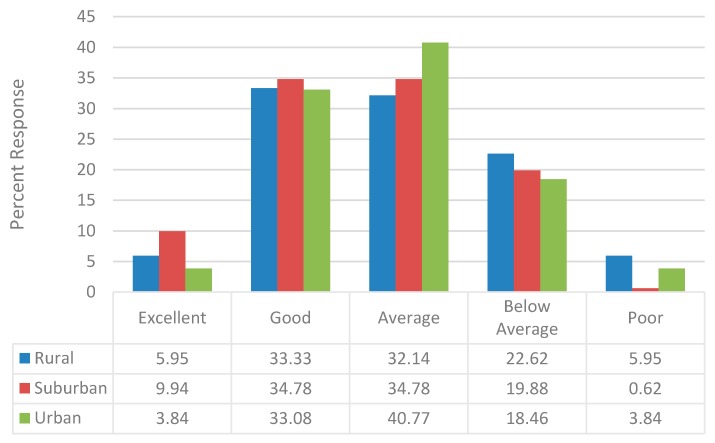
Pharmacy Financial Performance since 2006.

**Figure 2:**
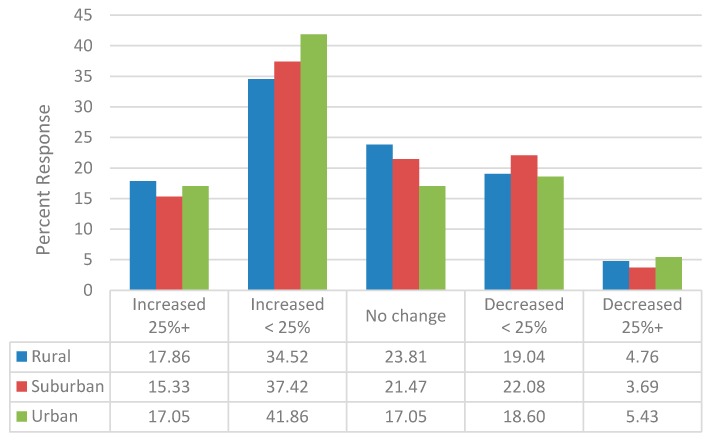
Change in Prescription Volume since 2006.

Less than half of the respondents (40.4%) indicated that the financial performance of their pharmacy since 2006 has been either excellent or good and 22.7% reported that it was either below average or poor. There were no geographic differences in the percentage of respondents reporting the financial performance of their pharmacy as excellent or good (rural: 39.3% suburban: 44.7%; urban: 36.9%; *P* = 0.3842), though a higher percentage of those practicing in rural locations reported a below average or poor financial performance (28.6% vs. 20.5% and 22.3%, respectively; *P* = 0.3534 (Figure 1)). Nevertheless, a majority (54.9%) reported an increase in the volume of prescription dispensed since 2006 (Figure 2). Over the two year period prior to the survey completion (years 2011 to 2013), a slightly lower percentage (41.5%) indicated an increase in prescription volume dispensed. The change in prescription volume since 2006 did not vary based upon geographic location.

**Figure 3:**
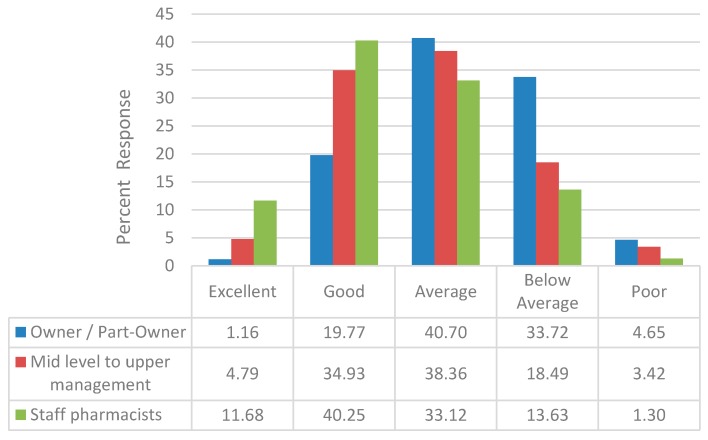
Pharmacy financial performance since 2006, stratified by employment role.

Pharmacists practicing at different capacities viewed the financial performance of their pharmacies since 2006 differently. Owners and part-owners of community pharmacies were less likely to view their pharmacies financial performance as excellent or good (20.9%, Figure 3) compared to mid to upper level pharmacy managers and staff pharmacists (39.7% and 51.9%, respectively; *P* = 0.0001). Several factors influenced the likelihood that the respondents either reported that the financial performance of their pharmacy as either excellent or good. Compared to pharmacists who practiced in an independent location, pharmacists who practiced in a ‘chain or other location’ were almost twice as likely to report the financial performance of their pharmacy as excellent or good. Pharmacists who practiced in pharmacies that received prescriptions electronically were less likely to report that the financial performance of their pharmacy as excellent or good as opposed to pharmacists who practiced in pharmacies that received none of their prescriptions electronically (Table 2). Pharmacists who practiced in pharmacies that dispensed more than 300 prescriptions per weekday were more than 3 times more likely to report that the pharmacy’s financial performance since 2006 as excellent or good as opposed to pharmacists who practiced in pharmacies that dispensed less than 100 prescriptions per weekday (Table 2).

The vast majority of respondents (91.7%) reported that they dispensed 90-day supply of medication at least some of the time, which may have accounted for some of the slowing in the volume of prescriptions dispensed. Work-flow factors imparted by Part D may also have affected pharmacy performance; 27.8% of the pharmacists reported that at least 40% of the Part D prescriptions they received were switched at the point of dispensing and 4.7% reporting that at least 70% were switched at the point of dispensing, creating work-flow disruption and costing time and money.

Although less than half of the owners and part-owners (44.7%) indicated that they were considering selling their pharmacy, nearly all (94.1%) reported that their decision to sell was due to the financial pressure exerted by Part D (Table 3). Despite this attestation, the decision to sell did not appear to be significantly related to the financial performance of the pharmacy since Part D inception in 2006, the volume of prescriptions dispensed, the volume or prescription dispensed in the last two years prior to the survey completion, or the dispensing of 90-day supplies of prescriptions. No demographic variables exerted a statistically significant relationship on respondents who were considering the selling their pharmacy with one exception: work status. Almost half (48.1%) of the owner or part-owners who were working full-time were considering selling their pharmacy, as opposed to none of the owners or part-owners working part-time (χ^2^ = 6.01, *P* = 0.0142). Rural pharmacy owners and part-owners were least likely to report considering the sale of the pharmacy (31.3% vs. 40.0% of suburban and 51.1% urban owners and part owners), despite being more likely to report a below average or poor financial performance for the pharmacy since 2006 (75.0% vs. 23.8% of suburban and 31.3% of urban owners and part owners (χ^2^ = 15.91, *P* = 0.0437).

### 3.3. MTM Services

Although two-thirds of the pharmacists reported providing MTM services at their primary practice sites, fewer (57.3%) reported that they were providing MTM services reimbursed by at least one Part D plan. While rural pharmacies were less likely to provide MTM (62.3%) compared to suburban and urban pharmacies (67.8% and 73.2%, respectively), rural pharmacies were more likely to provide MTM services reimbursed by at least one Part D plan (59.5%) than suburban pharmacies (54.0%) and similar to urban pharmacies (60.3%). None of these differences reached statistical significance. No relationships between providing MTM services and the financial performance of the pharmacy were observed (data not shown). Almost four-fifths of the pharmacists who were less than 40 years of age were providing MTM services, which was being reimbursed by at least one Part D plan as opposed to half of the pharmacists who were over the age of 40 years (Table 4). A statistically significant relationship between increased volume of prescriptions being dispensed (64.9%) and providing MTM services which were reimbursed by at least one Part D plan was observed (Table 4).

### 3.4. Part D 2010 Updates

We asked 3 specific questions targeting Part D 2010 updates, two of which were specifically related to the financial performance of the pharmacy and the third was indirectly related. Even though 85.1% of the pharmacists reported that they thought different pharmacies received different reimbursement for the fulfillment of their Part D prescription medications, only 16.1% felt that the new Center for Medicare and Medicaid Services (CMS) reporting requirement (required to report the actual price paid to the pharmacy) would have a positive financial impact on community pharmacies. The majority (68.7%) reported that it would be much easier to provide MTM services due to opt-out enrollment system. No statistically significant relationships were observed between these update related questions and all other variables (data not shown).

## 4. Discussion

Based on a thorough literature review, we believe that this is the first nationwide study conducted in the United States to understand the impact of Part D on community pharmacy. Previous analyses were regional in scope and primarily focused on rural independent pharmacies [12,13,17,30]. Contrary to previous analyses, we found the financial performance of community pharmacies nationwide since 2006 has been mixed, with pharmacists practicing at chain locations nearly twice as likely to report a better financial performance for their pharmacy compared to those at independent locations. It is possible that survivors bias, due to the number of independent pharmacy closures from 2006 onwards [33,34], may be underestimating the magnitude of the difference in the changes in financial performance between chain pharmacies and independent pharmacies since the introduction of Part D.

This study found that the financial performance of a community pharmacy is directly tied to the volume of prescriptions dispensed, as pharmacists who practiced in stores that dispensed 300 or more prescriptions per weekday were approximately 3 times more likely to report that the pharmacy’s financial performance as excellent or good as opposed to pharmacists who practiced in pharmacies that dispensed less than 100 prescriptions per weekday. The literature supports this direct relationship between volume and financial performance, albeit the magnitude of the effect being minor. An increase in annual prescription volume of 1000 prescriptions (approximately 20 prescriptions per week) has been associated with 0.4 percentage point increase in the likelihood of reporting a good or very good financial performance [35]. While Part D has contributed to an increase in prescription drug utilization nationwide [17], the increase has not been uniformly distributed; nearly one quarter of respondents reported a drop in prescription volume since 2006. Similar to a previous regional analysis [10,11], respondents reported that Part D created work-flow disruptions, costing both money and time.

Similar to previous research, we found that pharmacists practicing in rural locations were more likely to report a ‘below average’ or ‘poor’ financial performance since 2006 [12,17,30]. We also found a higher percentage of patients receiving Part D benefits in rural pharmacies and changes in reimbursement rates for these patients may have left these pharmacies more vulnerable to changes in reimbursement rates.

The community pharmacy industry in the United States has undergone tremendous change over last 10 years. Independent community pharmacies tend to be commonly located in rural areas, while chain community pharmacies are more concentrated in urban areas [36]. Further, as noted by Hoffman et al. (2016) rural pharmacies have declined in number between 2011 and 2016 [36]. This trend is expected to continue, as consumers can fill prescriptions at large chain pharmacies, independent pharmacies, mass merchandisers, supermarkets, warehouse stores and mail-order pharmacies. Retail pharmacy industry consolidation has resulted in a small number of large retailers controlling over 60 percent of total industry revenues [36] and the partial purchase of Rite Aid by Walgreens further consolidates industry dominance as it moves towards a duopoly controlled by Walgreens and CVS. Characteristic of mature industries, the pace of consolidation appears to be accelerating, with continued acquisition of smaller pharmacies by larger chains in an effort to expand geographic reach [36]. This consolidation is also fueled by pressure to improve financial performance in an environment characterized by “anemic” reimbursement rates [37]. The current industry trends lead to the conclusion that independent pharmacies will become fewer in number as large chain pharmacies assume greater industry dominance. Even that conclusion may appear null and void, given the most recent purchase of PillPack by Amazon [38], which has created a new market scenario for mail-order pharmacies. This purchase has sent shock waves through the industry; as reported by *Wall Street Journal*, Walgreens, CVS Health and Rite Aid (as well as the wholesalers) lost $22 billion in market value following this acquisition [39]. The implications for chain pharmacies have been immediately obvious, though implications for rural pharmacies are not as visible immediately. Financial performance pressures have stimulated the consolidation to streamline operations for cost savings and increase market shares that help to negotiate better drug price reimbursement rates with PBMs [25], a business strategy that is not available to the independent community pharmacy.

In 2015 there were approximately 22,160 independent community pharmacies, with 1800 as the sole provider in their rural community [40]. This is characteristic of past research reporting that counties within the United States with more lower-income and elderly residents had a higher proportion of independent community pharmacies [41]. Community pharmacies have been under increasing financial pressure due to the complexity of working with Part D plans, low reimbursement rates and lag in payments, so much so that their future viability as sole retail providers was in question [42]. This is important, as independent community pharmacies have been relied upon by consumers in underserved rural areas and the inner city [43].

Given this unsatisfactory situation, almost half of independent pharmacy owners and part-owners reported a desire to sell, the vast majority of whom cited financial pressure exerted by Part D as the reason for considering the sale. However, our analyses failed to connect an owner’s or part-owner’s decision to sell to the financial performance of the pharmacy, or the volume of prescriptions dispensed. When stratified by geographic location, rural pharmacy owners were most likely to report dispensing more than 300 prescriptions per weekday but were more likely than their urban or suburban counterparts to report the financial performance of the pharmacy since 2006 as below average or poor. As such, it is unclear as to the extent that financial pressures exerted by Part D continues to influence the interest in the sale or closure of independent pharmacies nationwide. Others have speculated that non-financial factors were leading to the sale or closure of rural independent pharmacies [44]. Our research supports others’ beliefs that the decision to continue to operate an independent pharmacy is based upon the owner’s perception of their financial position rather than the actual financial performance of the pharmacy [35]. These findings indicate a further need to conduct additional research to understand the challenges of owning and operating an independent community pharmacy independent of financial performance.

More than half of the pharmacists reported providing MTM services at their pharmacy, and these pharmacies were more likely to report an increase in prescription volume compared to those that did not. Nonetheless, the provision of MTM services did not result in a better financial performance. Given the voluntary and optional nature of MTM, there must be a self-perceived need of beneficiaries to obtain and benefit from these services [45]. However, rural older adults face unique challenges in accessing MTM services [33] and find it more challenging to comprehend the complexities of Part D [46]. Additionally, the earlier successes of MTM do not appear to be resonating in current clinical practice. A recent systematic review and meta-analysis reported insufficient evidence to demonstrate MTM interventions on many outcomes, including drug therapy problems, adverse drug events, disease-specific morbidity, disease specific or all-cause mortality and impairments [47]. When compared to usual care, MTM interventions were somewhat successful in improving a few measures of medication-related problems and health care use and costs (lowered odds of hospitalization and hospital costs); however, MTM interventions failed to improve patient satisfaction and health related quality of life [48]. This finding is in contrast to earlier literature, where the MTM-style interventions of the Asheville and Hickory Projects were associated with improvements in outcomes for various chronic diseases while reducing total health care costs [48,49,50]. Two tenets of the Asheville and Hickory Projects—use of specially trained pharmacists and a reduction in prescription co-payment—are absent from most current MTM programs. In this study, the majority of respondents were older pharmacists with Bachelors in Science in Pharmacy as their terminal degree. Targeted training of this group of older pharmacists may lead to the realization of better MTM outcomes for both patients and pharmacy owners.

Similar to previous research demonstrating more service orientation amongst rural pharmacists [51], our study found similar percentages of both rural and urban pharmacists providing MTM services which were reimbursed by at least one Part D plan. However, we found that rural pharmacy owners and part owners were less likely than their urban and suburban counterparts to provide MTM services that were reimbursed by Part D plans. Given the disparities in disease burden among rural older residents, a high prevalence of chronic illnesses and shortage of primary health providers [51], we believe that pharmacists practicing in rural locations have an important role to play in terms of improving the overall situation for older rural adults and pharmacy owners and part owners in rural areas have an opportunity to be more engaged in the care of their Part D patients.

While this survey determined whether or not respondents were providing MTM services at their pharmacy, the volume of MTM services provided was not captured. Community pharmacies face several barriers to offering MTM services, including staffing issues, physical barriers within the pharmacy itself and a lack of dedicated space for patient care areas; these barriers may be more pronounced within independent community pharmacies [12]. It remains unclear as to whether these barriers have been addressed in the decade since the opportunity for pharmacies to offer MTM services through Part D were first offered; additional research on this topic would be warranted. 

There are several limitations to this research which merit mention. This study has a low unadjusted response rate, with a final response rate of 419. However, the response rate for this study was comparable to a recently published online survey which appeared in the *Journal of Managed Care Pharmacy* [52] and we believe can be considered representative of the population studied. Other limitations included the authors’ inability to contact the non-responders directly (as e-mail addresses were controlled by the third-party vendor) and the use of a mailing list that included both business and personal email accounts. Amongst the respondents, we received an over-representation of practicing pharmacists from urban independent locations; as such, these respondents were more likely to be owners or part-owners of the pharmacy and more likely to have been in practice for more than 15 years. Nonetheless, since the survey participants were selected randomly from a national database of pharmacists, we believe the sample adequately represents pharmacists practicing in various states and settings across the country. Compared to the American Association of Colleges of Pharmacy (AACP) recent national survey of the pharmacist workforce [53], our respondent sample matched up well for many demographic variables, including age (73% of the AACP respondents >40 years of age vs. 80% of our sample) and full-time work status (82% for both surveys excluding retired and unemployed). Due to the intentional over-sampling of recent graduates (within 1–3 years of graduation) within the AACP survey [53], our respondent sample differed in a few demographic variables, including an overrepresentation of males (60% vs. 47%), those practicing with a terminal BSPharm degree (70% vs. 52%) and individuals practicing in independent pharmacies (57% vs. 22%). Nonetheless, the marginal response rate and the low response rate from the west coast limit the generalizability of the results. As with any survey, the results of this study are subject to non-response bias (with the worst performing pharmacies since the introduction of Part D ceasing operations) and social-desirability response bias.

## 5. Conclusions

Though a majority of community pharmacist respondents reported an increase in volume of prescription dispensed since 2006, less than the majority reported that their pharmacy experienced a favorable financial performance during the same timeframe. The provision of MTM services was not related to better pharmacy financial performance. Nearly half of pharmacy owners or part-owners indicated that they were considering selling their pharmacy, with most reporting that their decision to sell was due to the Part D financial pressures. However, the decision to sell was not related to the change in financial performance since 2006 or the volume of prescriptions dispensed.

## Figures and Tables

**Table 1:** Respondent demographics and practice characteristics.

	Total (*n* = 419)	Rural (*n* = 84)	Suburban (*n* = 163)	Urban (*n* = 131)
Male	60.5%	53.6%	60.7%	64.6%
Age (>40 years)	80.4%	76.2%	81.5%	81.7%
Primary Region of Practice (via US Census Bureau designation)				
Northeast	16.6%	27.3%	16.9%	7.8%
Midwest	31.9%	35.1%	29.2%	35.9%
South	39.5%	28.6%	40.9%	44.5%
West	11.9%	9.1%	13.0%	11.7% *
Primary Type of Practice Site				
Independent (1 store to 3 stores)	56.6%	51.8%	49.4%	71.8%
Chain (≥4 stores) and Other §	43.4%	48.2%	50.6%	28.2% **
Work Status (Full-Time)	82.0%	82.1%	80.1%	83.9%
Work Status (Part-Time 30 h or less)	18.0%	17.9%	19.9%	16.1%
Terminal Degree				
Doctor of Pharmacy	26.9%	29.3%	25.5%	26.7%
Bachelor of Science	69.9%	64.6%	71.4%	71.8%
Other	3.2%	6.1%	3.1%	1.5%
Primary Role as a Pharmacist				
Staff Pharmacist	40.1%	40.5%	52.8%	22.1%
Pharmacist-in-Charge/Pharmacy Manager/Part of Upper Level Pharmacy Management (District Manager)/Other	37.9%	40.5%	34.4%	41.2%
Community Pharmacy Part-Owner/Owner	22.0%	19.0%	12.9%	36.6% ***
Years of community pharmacy practice as a Registered Pharmacist				
15 years or less	25.6%	36.9%	24.4%	19.2%
More than 15 years	74.4%	63.1%	75.6%	80.8% ****
Number of prescription dispensed in a typical day				
0 to 300/weekday	67.1%	71.4%	59.9%	73.8%
>300/weekday	32.9%	28.6%	40.1%	26.2%
>50% of patients enrolled in Medicare Part D	29.4%	38.6%	25.8%	28.2% *****
Percentage of prescriptions received electronically				
Zero	1.1%	0	0.6%	2.3%
1% to 25%	32.3%	27.4%	29.4%	38.9%
26% to 50%	44.4%	51.2%	45.4%	38.9%
>50%	22.2%	21.4%	24.5%	19.8%

Percentages do not always sum to 100% because of missing data; Number of responses to the item of interest (*n*) varies because of missing data; § other included hospital outpatient pharmacies, rehabilitation facilities and long-term care pharmacies; * χ^2^ = 16.63, *P* = 0.011; ** χ^2^ = 16.38, *P* = 0.0003; *** χ^2^ = 37.17, *P* < 0.00001; **** χ^2^ = 8.57; *P* = 0.0138; ***** χ^2^ = 7.24; *P* = 0.0268.

**Table 2:** Factors influencing financial performance of the pharmacy.

Factor	Odds Ratio	95% CI	*P* value
Primary Practice Site *	**1.84**	**1.10 to 3.08**	**0.0211**
Primary Practice location **			
Rural	0.75	0.40 to 1.42	0.3834
Suburban	0.86	0.50 to 1.49	0.5900
Number of years in community practice	0.64	0.38 to 1.07	0.0892
Percentage of Part D patients	0.88	0.53 to 1.46	0.6116
Percentage of Prescriptions Received Electronically ***			
**1% to 25%**	**0.06**	**0.00 to 0.80**	**0.0329**
26% to 50%	0.10	0.01 to 1.33	0.0812
**>50%**	**0.06**	**0.00 to 0.81**	**0.0343**
Primary Role ****			
Pharmacist-in-charge, Pharmacy Manager, Part of Upper Level Management and Other	0.65	0.39 to 1.09	0.1013
**Community Pharmacy Owner and Part-Owner**	**0.38**	**0.18 to 0.79**	**0.0090**
Prescription Volume †			
100 to 300 per weekday	2.08	0.78 to 5.54	0.1421
**301 to 500 per weekday**	**2.95**	**1.02 to 8.58**	**0.0465**
**>500 per weekday**	**3.30**	**1.08 to 10.02**	**0.0356**

† Reference category less than 100 prescriptions dispensed per weekday; * Dichotomized as independent pharmacy = 0; ** Reference category urban location; *** Reference category: 0% or None; **** Reference category: staff pharmacist. Bold rows indicate statistical significance. Subgroup Analyses: Pharmacy Owners & Part-Owners.

**Table 3:** Subgroup analyses: pharmacy owners & part-owners.

	All	Rural	Suburban	Urban
Primary region of practice *				
Northeast	13 (14.9%)	5 (38.5%)	5 (23.8%)	1 (2.1%)
Mid-West	26 (29.9%)	3 (23.1%)	8 (38.1%)	14 (29.2%)
South	42 (48.3%)	4 (30.8%)	7 (33.3%)	29 (60.4%)
West	6 (6.9%)	1 (7.7%)	1 (4.8%)	4 (8.3%)
Percent of prescriptions received electronically **				
None	2 (2.2%)	0	0	2 (4.2%)
1% to 50%	71 (79.8%)	14 (87.5%)	12 (57.1%)	42 (87.5%)
>50%	16 (18.0%)	2 (12.5%)	9 (42.9%)	4 (8.3%)
Pharmacy’s financial performance since 2006 ***				
Excellent	1 (1.2%)	0	0	1 (2.1%)
Good	17 (19.8%)	2 (12.5%)	3 (14.3%)	12 (25.0%)
Average	35 (40.7%)	2 (12.5%)	13 (61.9%)	20 (41.7%)
Below average	29 (33.7%)	10 (62.5%)	5 (23.8%)	13 (27.1%)
Poor	4 (4.7%)	2 (12.5%)	0	2 (4.2%)
Prescription volume dispensed (past 2 years prior to survey completion) ****				
Decreased	30 (33.3%)	8 (50.0%)	4 (20.0%)	15 (33.3%)
Increased	39 (43.3%)	7 (43.8%)	7 (35.0%)	20 (44.4%)
Remained the same	21 (23.3%)	1 (6.3%)	9 (45.0%)	10 (22.2%)
Provide MTM which is reimbursed by at least one Part D plan *****	55 (64.0%)	6 (37.5%)	13 (65.0%)	34 (75.6%)
Number of prescription dispensed in a typical day of practice ******				
0 to 300/weekday	63 (75.0%)	8 (50.0%)	16 (76.2%)	39 (83.0%)
>300/weekday	21 (25.0%)	8 (50.0%)	5 (24.8%)	8 (17.0%)
Considerations regarding the sale of the pharmacy				
Respondent considering sale of the pharmacy	38 (44.7%)	5 (31.3%)	8 (40.0%)	24 (51.1%)
The decision to sell is influenced by the financial pressure exerted by Part D	32 (94.1%)	5 (100%)	6 (75.0%)	20 (83.3%)
Considering sale and have identified a potential buyer	14 (36.8%)	1 (20.0%)	4 (50.0%)	8 (33.3%)
Another community pharmacy located within 1 to 10 mile radius of the pharmacy considered for sale	33 (86.8%)	5 (100%)	7 (87.5%)	20 (83.3%)

* χ^2^ = 16.51, *P* = 0.0113; ** χ^2^ = 13.53, *P* = 0.009; *** χ^2^ = 15.91, *P* = 0.0437; **** χ^2^ = 8.36, *P* = 0.0791; ***** χ^2^ = 7.56, *P* = 0.0228; ****** χ^2^ = 6.94, *P* = 0.0310.

**Table 4:** Provision of Medication Therapy Management (MTM) and reimbursement by Medicare Part D.

	Do You Provide MTM Reimbursed by at Least one Part D Plan at Your Primary Practice Site?	
	Yes	No
All	220 (57.3%)	164 (42.7%)
Geographic location		
Rural	50 (59.5%)	34 (40.5%)
Suburban	88 (54.0%)	75 (46%)
Urban	79 (60.3%)	52 (39.7%)
Age (≤40 years)	57 (26.3%)	17 (10.6%)
Age (>40 years) *	160 (73.7%)	143 (89.4%)
Years of community pharmacy practice **		
15 years or less	67 (31.0%)	29 (18.2%)
More than 15 years	149 (69.0%)	130 (81.8%)
For the past 2 years, volume of prescription dispensed has ***		
Increased	98 (48.3%)	53 (33.5%)
Remained the same	62 (30.5%)	55 (34.8%)
Decreased	43 (21.2%)	50 (31.6%)

* χ^2^ = 14.28, *P* = 0.0002; ** χ^2^ = 7.85, *P* = 0.0051; *** χ^2^ = 8.88, *P* = 0.0118.
